# Supplementary material for: Cloaking, trapping and superlensing of lamb waves with negative refraction
Source: Sci Rep. 2021 Dec 13;11:23901. doi: 10.1038/s41598-021-03146-6 (PMC8668893; doi:10.1038/s41598-021-03146-6)
Supplement: Supplementary file 1 — Supplementary Information. [file 41598_2021_3146_MOESM1_ESM.pdf]

Supplementary Information
Cloaking, Trapping and Superlensing of Lamb Waves with Negative Refraction

François Legrand, Benoît Gérardin, François Bruno, Jérôme Laurent,
Fabrice Lemoult, Claire Prada, and Alexandre Aubry*
Institut Langevin, ESPCI, PSL University, CNRS, 1 rue Jussieu, F-75005 Paris, France
* *Corresponding author (e-mail: alexandre.aubry@espci.fr)*

(Dated: December 5, 2021)

This document provides further information on: (*i*) the mapping of the plate thickness in each experimental device; (*ii*) the study of complementary bands; (*iii*) the coherent backscattering effect induced by an imperfect corner; (*iii*) the design of blind holes in the numerical study of the superlens.

S1. PLATES THICKNESS MAPPING

In order to evaluate the quality of the manufactured plates, a local measurement of the plate thickness should be performed. To that aim, a map of the ZGV resonance frequency (Fig. 2) is achieved by means of a heterodyne interferometer and a pulsed Nd:Yag laser whose wavelength is 1064 nm (Centurion, Quantel)[1].

For the cloaking plates, the ZGV frequency measured on the thick part of the reference plate (f^+_{zgv} = 2.888 MHz) is higher than the one measured on the complementary plate (f^+_{zgv} = 2.812 MHz). The reference plate is thus thinner by a factor 0.97. This difference implies a slightly different wave-length λ_c exhibited by the S_2 and S_{2b} modes at the crossing frequency in each plate (see Fig. 3 of the accompanying paper).

Figures S1a and b display the ZGV frequency across the reference and complementary plates normalized by their respective f^+_{zgv}. This quantity thus yields the thickness across the plate normalized by its value obtained from an average over the black dotted rectangle. In each case, the thickness ratio between the thin and thick parts of the plate ranges between 0.88 and 0.91. Note that, in the complementary plate, the object and anti-object are not strictly mirror from each other. This partly explains the lower cloaking performance obtained experimentally compared to the numerical prediction (see Fig. 3 of the accompanying paper).

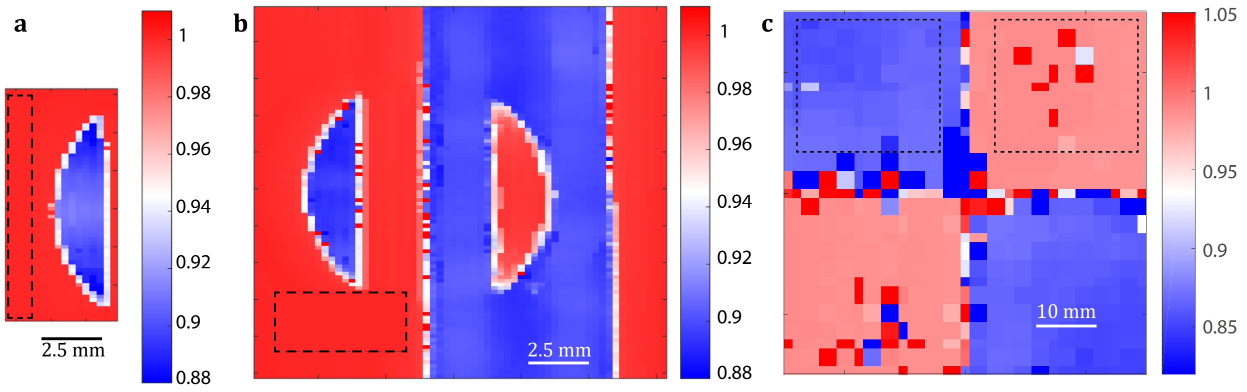

FIG. S1. **Maps of the plates thickness.** **a** Reference plate. **b** Complementary plate. **c** Double perfect corner plate. In each case, the measured thickness is normalized by its mean value (d_1 =1 mm) in the thick part.

A relative thickness map of the perfect corner device has been measured with the same technique. The result is displayed in Fig. S1c. The mean thickness ratio is d_2/d_1 = 0.86. This value implies a crossing frequency f_c = 3.46 MHz between the forward S_2 mode in the thick part and the backward S_{2b} mode in the thin part of the plate.

S2. COMPLEMENTARY BANDS

A negative refracting slab can be as a piece of negative space: It annihilates the diffraction of waves propagating over an equal thickness of opposite index. This striking property gave birth to the concept of complementary media. Two slabs of material of equal thickness and placed adjacent to one another annihilate each other if one is the negative mirror image of the other, the mirror being taken to lie on the interface between the two slabs.

The first configuration proposed by Pendry[3] consisted in associating two complementary bands of thickness L, an example of which is given by Fig. S2b. The phase accumulated by the wave during its travel through the first band is exactly compensated by the complementary band. At the output of this system, the transmitted wave-front is perfectly analogous to the incident one. The overall effect is as if a layer of space of thickness $2L$ had been removed from the experiment (Fig. S2c). This property holds whatever the incident wave-field. For the particular case of a point-like source, the complementary bands translate this source into a virtual one shifted by a distance $2L$ (Fig. S2b).

In this work, we have implemented this idea for guided elastic waves. The system studied here is a 1 mm-thick duralumin plate excited by a piezoelectric transducer that emits the forward S_2 mode as a cylindrical wave (Fig. S2a). The designed complementary bands consists in an arrangement of thickness steps over the plate. The positive and negative index areas corresponds to a plate thickness of 1 and 0.9 mm, respectively. At the crossing frequency and at each thickness step, there is a conversion between the forward S_2 and backward S_{2b} modes (see Fig. 2). The overall thickness $2L$ of the complementary bands is of 40 mm.

These devices are first investigated numerically using a FDTD code[2,4]. The simulation parameters are described in the Methods section of the accompanying paper. A normal displacement pulse is applied to the point source placed at a distance of 25 mm from the complementary bands. The normal displacement induced at the plate surface is recorded over a time length Δt = 110 μs. A spatio-temporal filter described in the Methods section is then applied to the recorded wave-field in order to isolate the S_2 and S_{2b} mode contributions. Figure S2d and e show the corresponding wave field and its phase at the crossing frequency f_c = 3.32 MHz. The incident wave is negatively refracted in the thinnest parts of the first complementary band, resulting in a strongly distorted wave-front at its output (x = 0 mm).

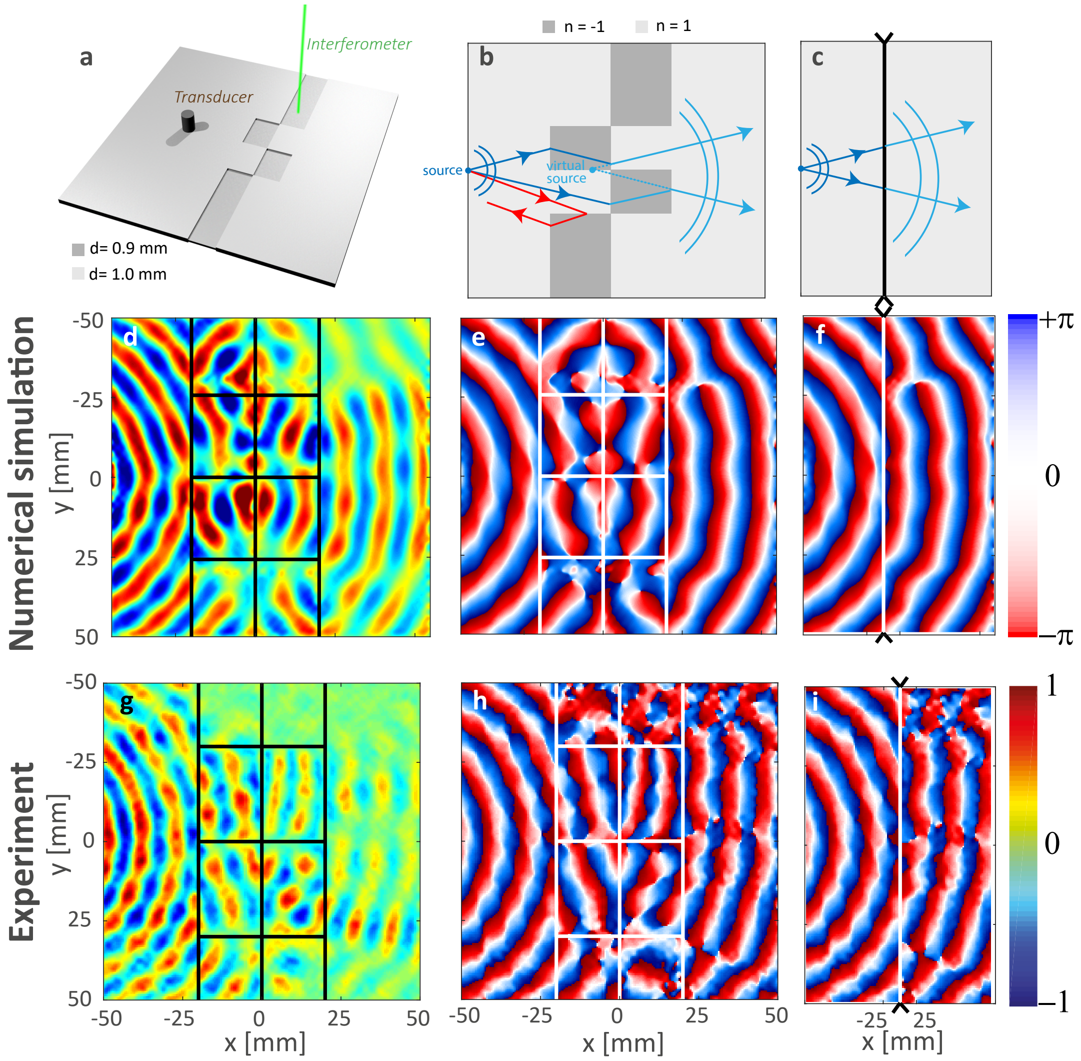

FIG. S2. **Cancelling wave diffraction over a region of space by means of complementary bands.** **a** Experimental configuration. **b** An alternative pair of complementary bands annihilates the effect of the other. Wave does not follow a straight line path in each layer, but the overall effect is as if a section of space thickness $2L$ were removed from the experiment. **c** The latter effect is highlighted by juxtaposing the wave-fronts upstream and downstream to the complementary bands. **d-f** Numerical simulation of the complementary bands (SimSonic3D software[2], www.simsonic.fr): Real part (**d**) and phase (**e**) of the normal displacement wave-field at the crossing frequency $f_c = 3.32$ MHz. (**f**) Juxtaposition of the wave-fronts at the input and output of the complementary bands as in **c**. **d-f** Experimental implementation of the complementary bands: Real part (**d**) and phase (**e**) of the normal displacement wave-field recorded at the crossing frequency $f_c = 3.37$ MHz. **f** Juxtaposition of the wave-fronts at the input and output of the complementary bands as in **c**.

The second complementary band then almost perfectly compensates for these phase distortions to give rise to a nearly cylindrical transmitted wave-front ($x > 20$ mm). The latter one is associated with a virtual source shifted by a distance $2L$ compared the *real* source location. It thus appears to be inside the complementary bands to a downstream observer. At the output of the device, wave propagates as if the space containing the complementary bands had disappeared. This effect is highlighted by juxtaposing the wave-fronts in the areas upstream and downstream to the complementary bands (Fig. S2c).

However, as shown by Fig. S2b, the magnitude of the wave-field is not perfectly homogeneous at the output. A shaded area occurs in the superior part of the plate for $y \in [-50; -25]$ mm. A first reason is the decrease of the conversion coefficient between the forward S_2 and backward S_{2b} modes for large angles of incidence[5]. In addition, a fraction of the incident wave is reflected by the corners between the positive and negative index areas (see for instance the red arrow in Fig. S2b). These spurious reflections induce an heterogeneous angular distribution of the transmitted wave-front across the complementary bands. This effect had already been noticed by Pendry[3] but, in an ideal case, those spurious reflections would be eliminated by destructive interferences between complementary corners via an evanescent coupling between them. In the present case, such a mechanism does not occur for Lamb waves and the reflections induced by the complementary corners give rise to a shadow zone behind the complementary bands.

Following this numerical study, the complementary band plate is manufactured on a duralumin plate by electrical discharge machining. The source is a piezoelectric transducer glued on the plate at 25 mm from the complementary

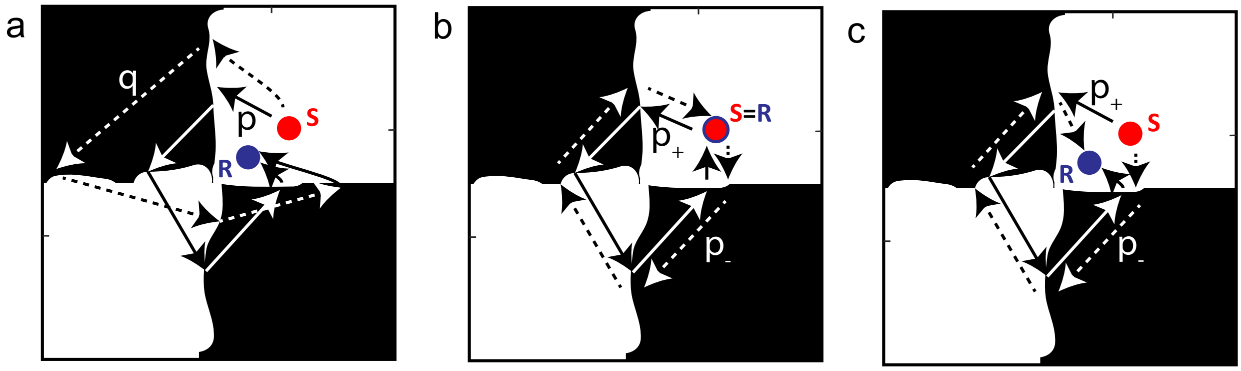

FIG. S3. **Coherent backscattering phenomenon at the vicinity of an imperfect corner**. **a** Examples of paths p and q that can undertake the wave between the source S and receiver R in presence of rough interfaces and/or index fluctuations. **b** Constructive interference between reciprocal paths p_+ and p_- when the source and receiver are at the same location. **b** This interference phenomenon vanishes when the receiver is beyond one half-wavelength from the initial source location .

bands. The normal displacement is measured at the plate surface using a photorefractive interferometer (see Methods of the accompanying paper). The wave-field filtered at the crossing frequency and its phase are shown in Fig. S2g and h, respectively. The crossing frequency f_c is here of 3.37 MHz and slightly differs from its theoretical value (f_c = 3.32 MHz). Negative index areas actually display a thickness of 0.89 mm instead of 0.9 mm. The recorded wave-field shows some difference compared to its numerical counterpart (Figs. S2d and e). Spurious reflections induced by thickness step corners, already highlighted by the numerical simulations in Fig. S2d, are here enhanced by an imperfect tailoring of such singularities. The angular distribution of the transmitted wave-field magnitude thus exhibits strong fluctuations. Nevertheless, the effect of the complementary bands is nicely highlighted by investigating the phase of the wave-field (Fig. S2e). The juxtaposition of the wave-fronts at input and output of the complementary device displayed in Fig. S2f shows the mutual compensation of the phase accumulated by the wave in each complementary band.

The experimental implementation of these complementary bands are a first step towards the ability of cloaking a region of space by its anti-object demonstrated in the accompanying paper (Fig. 3). This experiment highlights the importance of a precise tailoring of negative corners. Despite their strong dependence on manufacturing imperfections, such singularities can be leveraged for wave trapping. This is illustrated by the experimental implementation of the double negative corner in the accompanying paper (Figs. 4 and 5).

S3. COHERENT BACKSCATTERING INDUCED BY AN IMPERFECT CORNER

In a double negative corner, disorder can be induced by the roughness of the interfaces between positive and negative index area and/or by index fluctuations in each of these area (see Supplementary Section S1). In such conditions, the wave-field $\psi(S,R)$ recorded by the receiver R following the emission of a pulse by a point source S can still be decomposed as a sum of partial waves ψ_p, each one being associated with a particular path p between S and R (see Fig. S3a):

$$\psi(S,R) = \sum_p \psi_p \tag{S1}$$

The corresponding mean intensity $\langle|\psi(S,R)|^2\rangle$ can be decomposed as the sum of two terms:

$$\langle|\psi(S,R)|^2\rangle = \underbrace{\left\langle \sum_p |\psi_p|^2 \right\rangle}_{\text{Incoherent intensity}} + \underbrace{\left\langle \sum_p \sum_{q\neq p} \psi_p \psi_q^* \right\rangle}_{\text{Coherent intensity}} \tag{S2}$$

where the symbol $\langle\cdots\rangle$ account for an ensemble average over disorder configurations. The first term in Eq. S2 is said incoherent and represents the self-interference of each partial wave with itself. The second term is said to be coherent and accounts for the interference between partial waves following a different trajectory between S and R (see, for instance, paths p and q in Fig. S3a). In presence of disorder, these partial waves can be assumed as uncorrelated: The coherent intensity is thus equal to zero. However, when source and receiver coincide, the two reciprocal wave packets are in phase and interfere constructively, independently of the particular realization of disorder (see, for instance, paths p_+ and p_- in Fig. S3b). Such constructive interferences will thus persist after ensemble averaging and an intensity enhancement is observed at the initial source location even in presence of disorder. If source S and

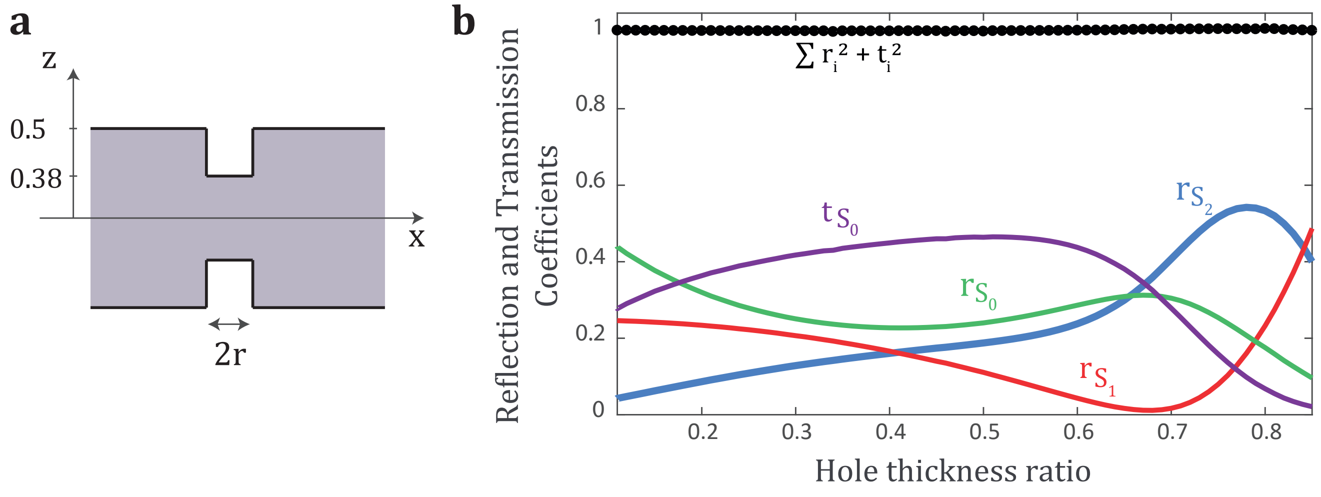

FIG. S4. **Design of the metalens**. **a** Geometry of a single blind hole. **b** Theoretical reflection and transmission coefficients of the incident S_2 mode as a function of the thickness ratio in a 1 mm thick duralumin plate at the frequency $f = 3.31$ MHz.

receiver R do not coincide, the phases of the two reciprocal waves are not necessarily equal and will depend on the particular realization of disorder (see Fig. S3c). The coherent backscattering effect is thus expected to decrease with source–receiver distance.

S4. DESIGN OF THE METALENS

The metalens used in the superlens simulation is made of blind holes[6–8] that are known as efficient sub-wavelength resonators for Lamb waves. Their geometry displayed in Fig. S4a has been designed to ensure various properties. First, the holes are symmetrical to avoid any conversion into anti-symmetric Lamb modes. Secondly, their radius and the distance between them are set at 2 mm and 1 mm, respectively. These values are small compared to the S_2-mode wavelength ($\lambda_c = 12.5$ mm) in order to efficiently scatter the evanescent components of the source. Finally, the thickness ratio of the blind holes has been fixed to maximize the reflection of the S_2-mode into itself. To that aim, we use the semi-analytical model previously developed to study negative refraction at a thickness step[5]. Figure S4b shows the reflection and transmission coefficient of the incident S_2 mode as a function of the thickness ratio displayed by the blind hole. A maximum reflection coefficient is obtained for a ratio of 0.76. Note that this semi-analytical model is valid for a plane interface. Its application to holes whose dimensions are lower than the wavelength is highly questionable. Nevertheless, the obtained value leads to a significant amount of scattering of the incident S_2 mode into itself by the blind holes (see Fig. 6 of the accompanying paper).

[1] C. Prada, O. Balogun, and T. W. Murray, Appl. Phys. Lett. **87**, 194109 (2005).
[2] E. Bossy, M. Talmant, and P. Laugier, J. Acoust. Soc. Am. **115**, 2314 (2004).
[3] J. B. Pendry, Contemp. Phys. **45**, 191 (2004).
[4] E. Bossy, "Simsonic, a FDTD simulation freeware," (2003), www.simsonic.fr.
[5] F. Legrand, B. Gérardin, J. Laurent, C. Prada, and A. Aubry, Phys. Rev. B **98**, 214114 (2018).
[6] O. Xeridat, *Etude expérimentale de la propagation, de la diffusion et de la localisation des ondes de Lamb*, Ph.D. thesis, Université de Nice (2011).
[7] M. Dubois, *Contrôle des ondes de flexion dans les plaques*, Ph.D. thesis, Université Paris Diderot-Paris 7-Sorbonne Paris Cité (2014).
[8] M. Dubois, C. Shi, X. Zhu, Y. Wang, and X. Zhang, Nat. Commun. **8**, 14871 (2017)
